# Prolonged mechanical ventilation–induced neuroinflammation affects postoperative memory dysfunction in surgical mice

**DOI:** 10.1186/s13054-015-0882-0

**Published:** 2015-04-10

**Authors:** Chang Chen, Zongze Zhang, Ting Chen, Mian Peng, Xing Xu, Yanlin Wang

**Affiliations:** Department of Anesthesiology, Zhongnan Hospital, Wuhan University, East Lake Road, Wuhan, 430071 Hubei China

## Abstract

**Introduction:**

Patients undergoing surgery frequently develop neuropsychological disturbances, including cognitive decline or memory impairment, and routine clinical procedures such as mechanical ventilation (MV) may affect acute-phase brain outcome. We aimed to investigate the effect of the prolonged MV on postoperative memory dysfunction in surgical mice.

**Methods:**

Male C57BL/6 mice were randomly divided into the following three groups: (1) The control group (group C) comprised anesthetized, unventilated animals; (2) the surgery group (subgroups S1h, S3h and S6h) was unventilated animals that underwent surgery under general anesthesia; and (3) the MV group (subgroups MV1h, MV3h and MV6h) was made up of animals under MV for 1 hour, 3 hours or 6 hours after surgery. Separate cohorts of animals were tested for memory function with fear conditioning tests or were killed at 6 hours, 1 day or 3 days postsurgery or post-MV to examine levels systemic and hippocampal interleukin (IL)-1β, IL-6 and tumor necrosis factor α (TNFα), and assessed synaptic structure and microglial activation. Nuclear factor κB (NF-κB) p65, cytochrome *c*, cleaved caspase-3 and cleaved poly(ADP-ribose) polymerase (PARP) activation were analyzed by Western blotting.

**Results:**

The MV6h group showed increased CD11b-immunopositive cells, synapse degeneration, cytochrome *c* release, cleaved caspase-3 and cleaved PARP-1 activation after surgery, as well as a decrease in freezing time after surgery. At 6 hours and 1 day post-MV, MV6h increased NF-κB activation and levels of systemic and hippocampal IL-1β, IL-6 and TNFα after surgery.

**Conclusions:**

Prolonged MV after surgery further aggravates cognitive decline that may stem from upregulation of hippocampal IL-1β, IL-6 and TNFα, partially via activation of gliocytes in the surgical mouse hippocampus.

## Introduction

Mechanical ventilation (MV) is often a lifesaving intervention in critically ill patients and is frequently used in patients under general anesthesia during surgical operation [1]. It is known that the need for MV has been implicated in the development of delirium [2]. In epidemiological studies, intubation and positive pressure ventilation increase the incidence of delirium by up to 74% to 83% compared with 20% to 48% in nonintubated patients [3]. Moreover, critical care patients who undergo long-term MV show distinctive neurological impairment, including memory and cognitive decline [4].

Many studies have been focused on the lung–brain axis with the purpose of determining which factors implicated in acute lung injury [5] and in its ventilatory management can give rise to the appearance of cognitive alterations [6]. However, we know remarkably little about the mechanisms through which damage to remote organs can reach the brain. There is evidence in ventilated animals that MV triggers hippocampal apoptosis by vagal and dopaminergic pathways [7,8]. During the past few years, an increasing amount of evidence has supported the view that the excessive release of proinflammatory cytokines, including tumor necrosis factor α (TNFα), interleukin (IL)-1β and IL-6, is involved in cognitive impairment after surgery [9]. However, the pathogenesis of MV-increased, surgery-induced cognitive impairment is poorly understood, including early neurological effects related to MV and the central nervous system (CNS) response to systemic inflammation.

The main objectives of the present study were to investigate the effect of prolonged MV on neuroinflammation in a murine model of MV following orthopedic surgery and to examine the extent to which MV may aggravate acute memory dysfunction. Thus, we measured morphological changes of microglial reactivity induced by MV; the levels of IL-1β, IL-6 and TNFα in plasma and hippocampus; nuclear factor κB (NF-κB) p65 expression; and the hallmark of apoptotic cascades. In addition, the effects of long-term MV on postoperative memory dysfunction in surgical mice were evaluated.

## Materials and methods

### Ethical approval

The experiments were performed in accordance with a protocol approved by the animal use and care committee of Wuhan University, Hubei, China, and in accordance with the National Institutes of Health guidelines. This study was approved by the animal ethics committee at the Zhongnan Hospital and Research Centre, Hubei, China.

### Animals

Normal male C57BL/6 wild-type mice weighing between 20 and 25 g, 6 to 8 weeks of age, were purchased from medical the experimental animal center of Hubei province. The animals were housed in individual cages in a temperature-, humidity- and light-controlled room (12-hour light-dark cycle) and were acclimated to these conditions for at least 7 days prior to use in experiments. Under aseptic conditions, mice were subjected to an open tibial fracture of the left hind paw with an intramedullary fixation [10]. Briefly, mice received general anesthesia with 2% isoflurane, and analgesia was achieved with buprenorphine 0.1 mg/kg administered subcutaneously, immediately after anesthetic induction and before surgical insult. A midline incision was performed on the left hind paw, and a 0.38-mm pin was inserted into the intramedullary canal, the periosteum was stripped and an osteotomy was performed. Temperature-controlled, light-emitting diode shower lights were used to maintain body temperature at 37 ± 0.5°C. The entire procedure, from induction of anesthesia to the end of surgery, lasted 12 ± 5 minutes, and then the mice were suspended at a 45° angle, with a light source in the neck area and a homemade metal laryngoscope adjusted to provide the best visualization of the vocal cords. A 22-gauge venous catheter with a needle core was inserted 3 mm into the trachea of the mice [11]. Then, with the needle core pulled out, the catheter was connected to the ventilator (TOPO; Kent Scientific, Torrington, CT, USA). The respiratory rate was set at 100 breaths/min, and the pressure control mode was set with a peak inspiratory pressure of 12 to 15 cmH_2_O. The fraction of inspiration oxygen (FiO_2_) was kept at 0.5. Arterial oxygen saturation was measured noninvasively using a MouseOx pulse oximetry system (Starr Life Sciences, Oakmont, PA, USA) during anesthesia. After surgery, the mice randomized to the MV group received 1.2% isoflurane/oxygen (FiO_2_ =0.5) for 1 hour, 3 hours or 6 hours, and the S group was placed in an anesthetizing chamber flushed with 1.2% isoflurane/oxygen (FiO_2_ =0.5) for 1 hour, 3 hours or 6 hours and did not undergo tracheal intubation. Anesthetic and oxygen concentrations were measured continuously (GE Healthcare, Wauwatosa, WI, USA), and the temperature of the anesthetizing chamber was controlled to maintain rat body temperature at 37 ± 0.5°C [12,13]. After MV, the anesthetics were discontinued, and all animals were allowed to recover for 20 minutes in a box flushed with 100% oxygen and then placed in their home cages.

### Experimental protocol

C57BL/6 mice were randomly divided into the following three groups: a control (C) group (group C1h, C3h and C6h), a surgery (S) group (groups S1h, S3h and S6h) and a MV group (groups MV1h, MV3h and MV6h). In the control group, mice that did not undergo surgery received anesthesia/analgesia alone and were put in the anesthesia chamber with isoflurane for 1 hour, 3 hours or 6 hours and maintained on spontaneous breathing. In the surgery group, surgery consisted of an open tibial fracture with intramedullary fixation in aseptic conditions under general anesthesia with isoflurane and buprenorphine, and mice were kept on spontaneous breathing and then placed in the anesthesia chamber with isoflurane for 1 hour, 3 hours or 6 hours. In the MV group, after the same surgery, 1.2% isoflurane was administered to maintain anesthetic levels during the MV 1-hour, 3-hour and 6-hour procedures. Animals were trained 24 hours prior to surgery using a fear conditioning (FC) protocol and assessed in their training environment and in a novel context 6 hours, 1 day and 3 days after treatment. Blood was collected by cardiac puncture, and the hippocampus was removed at 6 hours, 1 day and 3 days postsurgery or post-MV. Plasma and hippocampal IL-1β, IL-6 and TNFα were measured by enzyme-linked immunosorbent assay. Fixed brains were collected for immunohistochemical for microglial activation using CD11b and for ultrastructure changes of synapses by transmission electron microscopy (TEM). Western blot analysis was performed for NF-κB p65 protein expression, cytochrome *c* (Cytc) release and cleaved caspase-3 and cleaved poly(ADP-ribose) polymerase (PARP)-1 activation.

The animals were tagged and randomly allocated to each group before any treatment or procedure. Researchers were blinded to the group assignment, which was revealed only after the analysis phase.

### Fear conditioning tests

**Figure 1:**
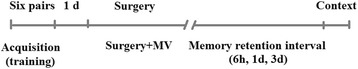
**Assessment of efficiency of consolidation of memory using a contextual fear conditioning protocol.** Mice were frightened by an aversive stimulus, in this case tone and electrical foot shock stimulus, to acquire fear memory (acquisition). One day prior to surgery (n =36) or mechanical ventilation (MV; n =36), animals were trained for fear conditioning. At 6 hours, 1 day and 3 days after those treatments, memory was reassessed by measuring the period of time during which the animal became involuntarily immobile when reintroduced to the aversive context.

Freezing behavior is an indicator of aversive memory that is measured when subject mice are reexposed to the conditional stimulus. For this study, we used a previously published paradigm [14]. The FC paradigm consists of a training phase prior to surgery and an evaluation phase after surgery or MV when memory is assessed. Briefly, 1 day prior to surgery or MV, control (n =12), surgery (n =36) and MV (n =36) animals were trained for FC to learn the task and establish long-term memory. Mice were allowed to familiarize themselves with the surroundings (context) for 120 seconds, followed by a 20-second, 70-dB tone (conditional stimulus) and then a delay of 25 seconds. This contextual interval was terminated by an unconditional stimulus, a 0.70-mA electrical foot shock for 2 milliseconds. The pairs of conditional–unconditional stimuli were separated by random intervals from 45 to 60 seconds, which was the intertraining interval. The intertraining interval allowed the mice to disengage from the process of association before a new set of stimuli was introduced. After six pairs of conditional–unconditional stimuli, the mice learned the association and established long-term memory. After surgery or MV, mice were placed back in the original conditioning chamber, where no tone or shock was presented, to assess recall of context and/or environment 6 hours, 1 day and 3 days after surgery, exposure to MV or neither (Figure 1). Our measure of associative learning was the percentage of time spent not moving (percentage freezing time). Behavior was captured with an infrared video camera (Sony Corporation, Tokyo, Japan).The observer was unaware of the treatment received by mice at the time of the behavioral assessment.

### Enzyme-linked immunosorbent assay

Blood was collected into heparin-coated syringes at 6 hours, 1 day and 6 days post-MV after thoracotomy under terminal isoflurane anesthesia. Samples were centrifuged at 3,400 rotations per minute for 10 minutes, and plasma was collected and stored at −80°C until assayed. Hippocampal tissues were homogenized on ice in 20 mM Tris-HCl buffer (pH 7.3) containing protease inhibitors. Homogenates were centrifuged at 10,000 × *g* for 10 minutes at 4°C. The supernatant was then ultracentrifuged at 150,000 × *g* for 2 hours. Plasma and hippocampal tissue IL-1β, IL-6 and TNFα were measured using commercially available enzyme-linked immunosorbent assay kits according to the manufacturer’s instructions (Santa Cruz Biotechnology, Santa Cruz, CA, USA). All samples were assayed in duplicates. The readings were normalized to the amount of standard protein.

### Immunofluorescence

At 6 hours, 1 day and 3 days after surgery (n =12) and MV (n =12), the animals were anesthetized with isoflurane. The thoracic cavities were opened and perfused intracardially with 40 ml of cold saline, followed by 4% paraformaldehyde. Then, the brain was rapidly taken out, postfixed in 4% paraformaldehyde at 4°C, embedded in optimal cutting temperature tissue-freezing medium and sectioned for immunofluorescence. Tissue sections were blocked in 5% bovine serum albumin. After three washes in phosphate buffer, the sections were incubated with a mouse monoclonal anti-CD11b antibody (1:200; Abcam, Cambridge, UK) at 4°C overnight. After several washes, the sections were incubated with a goat anti-mouse immunoglobulin G secondary antibody (1:100; Jackson ImmunoResearch Laboratories, West Grove, PA, USA) for 1 hour in the dark. After rinsing with phosphate-buffered saline, the sections were mounted on slides with Hoechst 33342 dye for 5 minutes. After washes, the sections were subsequently observed, and images were acquired using an imaging system equipped with a fluorescence microscope (Olympus, Tokyo, Japan). For each animal, the number of CD11b-positive cells in three hippocampal subregions—CA1, CA2 and CA3—were estimated from photomicrographs with a counting frame size of 0.4 mm^2^. The number of CD11b-positive cells per square millimeter were counted in three counting frames per region (total of nine frames per animal) by using Image J software (National Institutes of Health, Bethesda, MD, USA), and the numbers of cells in the three frames per region were then averaged.

### Transmission electron microscopy

The animals were anesthetized with isoflurane. The thoracic cavities were opened and perfused intracardially with ice-cold saline, followed by perfusion with 4% paraformaldehyde fixative for 10 minutes. Coronal sections were cut 150 μm thick on a vibrotome, and the area of the CA1 pyramidal layer and stratum radiatum was dissected out. Briefly, microdissected areas were washed in 0.1 M/L sodium phosphate buffer and postfixed at room temperature for 1 hour in 1% osmium tetroxide. Samples were then rinsed in ultrapure water, and tissue blocks were dehydrated at room temperature through graded ethanols from 30% to 100% for 10 minutes each, including 1% uranyl acetate in 70% ethanol for 40 minutes, and embedded in Epon epoxy medium (Momentive Specialty Chemicals/Hexion, Columbus, OH, USA). Twenty-four hours later, 120-nm sections were cut with an ultramicrotome (DuPont, Wilmington, DE, USA) and stained with 4% uranyl acetate for 20 minutes and 0.5% lead citrate for 5 minutes. Ultrastructural changes of synapses in the CA1 were observed under a Hitachi HT7700 TEM microscope (Hitachi, Tokyo, Japan) and subsequently processed using Adobe PhotoShop software (Adobe Systems, San Jose, CA, USA). Synaptic structure in CA1 was analyzed in at least 20 images per mouse (n =3). Measurement of postsynaptic density (PSD) areas, width of synaptic cleft and number of vesicles were included only if synaptic terminal profiles were clearly visible, and these were performed using ImageJ software. The identities of images were coded and revealed to the observer only after the data analysis was complete.

### Nuclear protein extraction

Cytoplasmic and nuclear proteins were prepared using a nuclear and cytoplasmic protein extraction kit (KeyGen Biotech, Nanjing, China) following the manufacturer’s instructions. Hippocampal tissues were homogenized on ice and resuspended in the cytoplasmic protein extraction reagent, then centrifuged at 12,000 × *g* at 4°C for 5 minutes. The supernatant was cytoplasmic protein, and the pellet was resuspended in the nuclear protein extraction reagent and centrifuged at 12,000 × *g* at 4°C for 10 minutes. The supernatant was nuclear protein. The nuclear protein was subjected to Western blot analysis.

### Western blot analysis

The animals were killed at 6 hours, 1 day or 3 days post-MV. The hippocampus, including CA1 and the dentate gyrus field, was homogenized on ice using immunoprecipitation buffer (10 mM Tris-HCl, pH 7.4, 150 nM NaCl, 2 mM ethylenediaminetetraacetic acid and 0.5% Nonidet P-40) plus protease inhibitors (1 μg/ml aprotinin, 1 μg/ml leupeptin and 1 μg/ml pepstatin A). The lysates were collected and then centrifuged at 13,000 × *g* at 4°C for 30 minutes. Protein concentrations of samples were determined using a bicinchoninic acid protein assay (Beyotime Institute of Biotechnology, Haimen, China). To determine apoptosis in the hippocampus after MV and surgical exposures, Cytc, caspase-3 and PARP-1 levels were examined. Briefly, the blots were incubated with the following monoclonal antibodies, respectively: anti-Cytc at 1:1,000 dilution, anti-cleaved caspase-3 at 1:2,000 dilution and anti-cleaved PARP-1 at 1:500 dilution. All antibodies were purchased from Cell Signaling Technology (Danvers, MA, USA). Next, samples were probed with horseradish peroxidase–conjugated secondary antibody. To study the effect of MV on NF-κB in hippocampal tissues after surgery, we examined the protein levels of NF-κB p65 (1:500 dilution; Santa Cruz Biotechnology). Images were acquired by using a CanonScan LiDE110 scanner (Canon, Melville, NY, USA) and analyzed using the AlphaImager EP imaging system (NatureGene, Beijing, China). The results for NF-κB p65 were normalized to those of total histone H3 (Cell Signaling Technology Inc., Beverly, MA, USA) H3. The data of Cytc, cleaved caspase-3 and cleaved PARP-1 were normalized to those of β-actin. The results are expressed as relative density.

### Statistical analysis

Statistical analysis was performed with IBM SPSS software (version 19.0; IBM, Armonk, NY, USA). The results are expressed as mean ± standard error of the mean. Statistical analysis was performed with analysis of variance followed by the Student-Newman-Keuls multiple-comparisons test for numerical data. Differences between two groups were assessed with Student’s *t*-test for data normally distributed and with the Mann–Whitney rank-sum test for data non-normally distributed, under the supervision of an expert statistician. Significance was set at *P* <0.05.

## Results

### Cognitive decline

**Figure 2:**
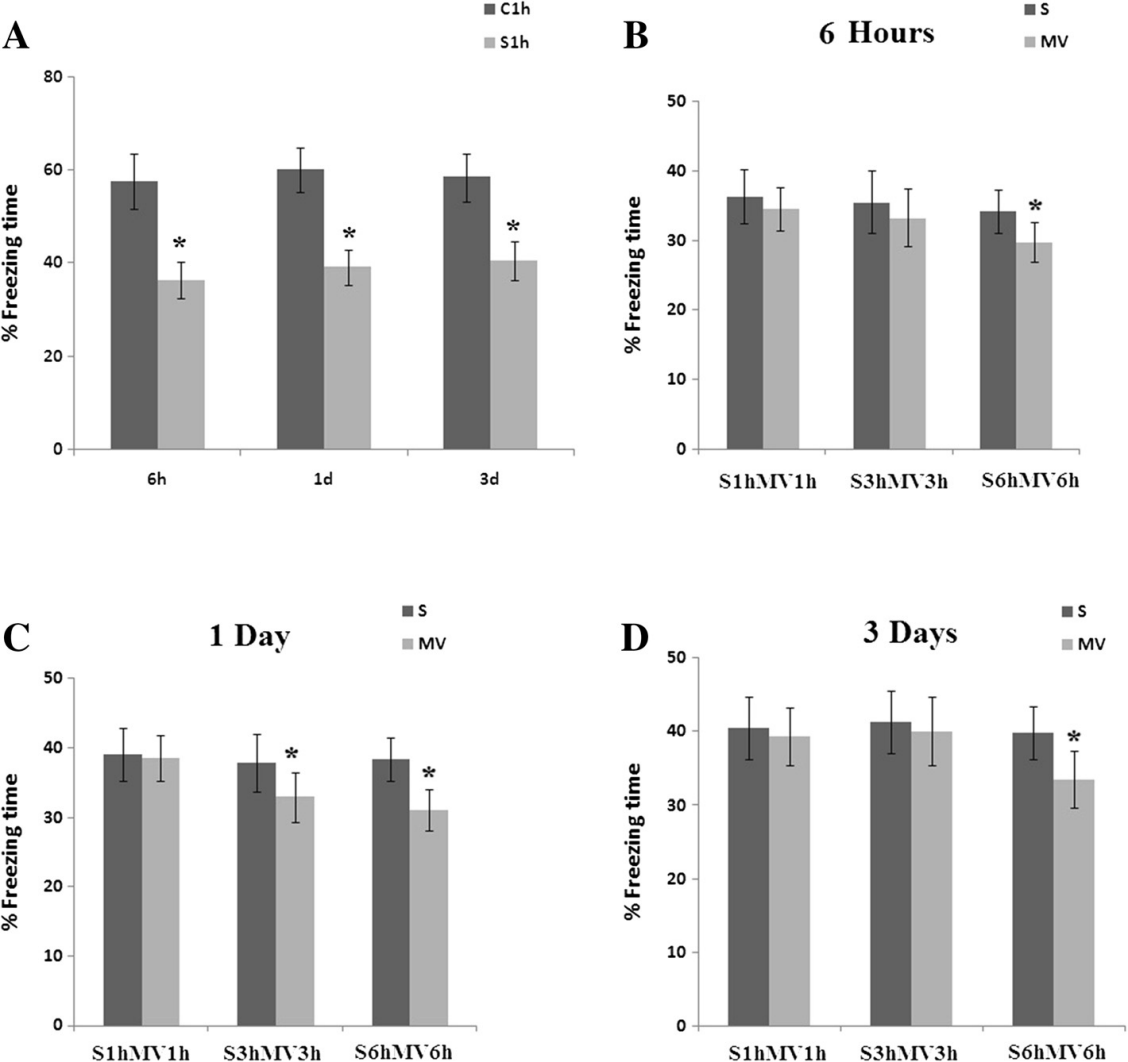
**Contextual fear conditioning responses after surgery followed by mechanical ventilation.** In the surgery group (S), surgery consisted of an open tibial fracture with intramedullary fixation in aseptic conditions under general anesthesia with isoflurane and buprenorphine, and then mice were placed in the anesthesia chamber with isoflurane for 1 hour, 3 hours or 6 hours. In the mechanical ventilation (MV) group, after the same surgery, isoflurane was administered to maintain anesthetic level during MV for 1 hour, 3-hour and 6-hour procedures. **(A)** Freezing time of C1h and S1h subgroups. **(B)** Percentage freezing time in the 6-hour groups. **(C)** Percentage freezing time on day 1. **(D)** Percentage freezing time on day 3. n =12/group. Values are mean ± standard error of the mean. *P <0.05 indicates significant differences.

During the preoperative training period, learning was similar in the MV groups and surgery groups (data not shown). Surgery significantly decreased the percentage of freezing time compared with the control group (Figure 2A). One hour exposure to MV after surgery failed to significantly affect freezing time when compared with surgical animals at any time point examined (*P* >0.05). However, after 6-hour exposure to MV, mice showed significantly reduced memory following surgery at 6 hours, 1 day and 3 days post-MV (*P* <0.05) (Figure 2).

### Inflammatory response

**Figure 3:**
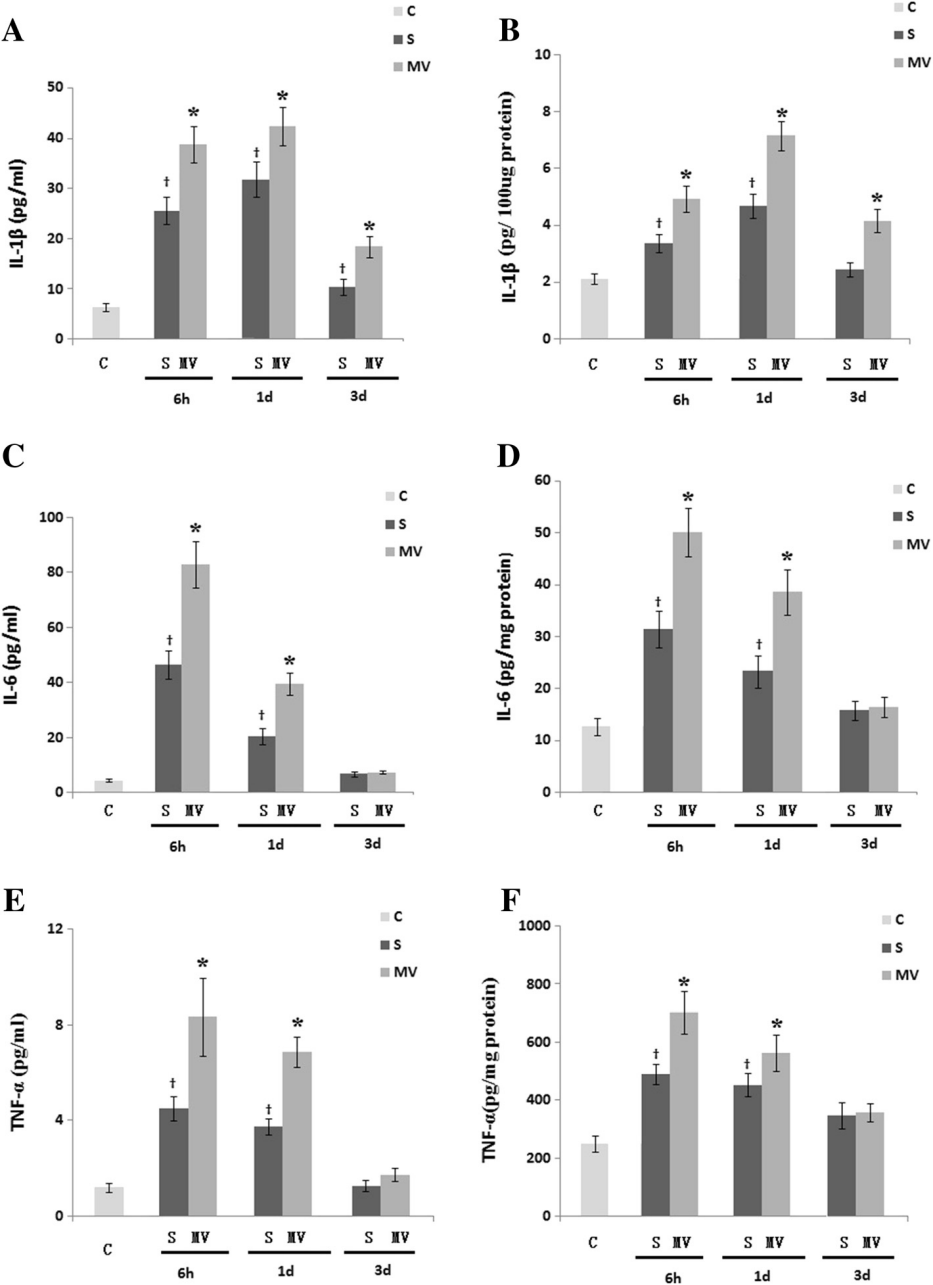
**Effects of mechanical ventilation on the levels of interleukin 1β, interleukin 6 and tumor necrosis factor α after surgery in the plasma and hippocampus.** Six-hour exposure to mechanical ventilation (MV) resulted in elevated plasma **(A,** **C and** **E)** and hippocampus **(B, D and** **F)** levels of interleukin (IL)-1β, IL-6 and tumor necrosis factor α (TNFα) at both 6 hours and 1 day after MV compared with the surgery group (S), as measured by enzyme-linked immunosorbent assay. Values are mean ± standard error of the mean. n =6/group. *P <0.05, compared with group S; ^†^ *P* <0.05 compared with the control group (C).

**Figure 4:**
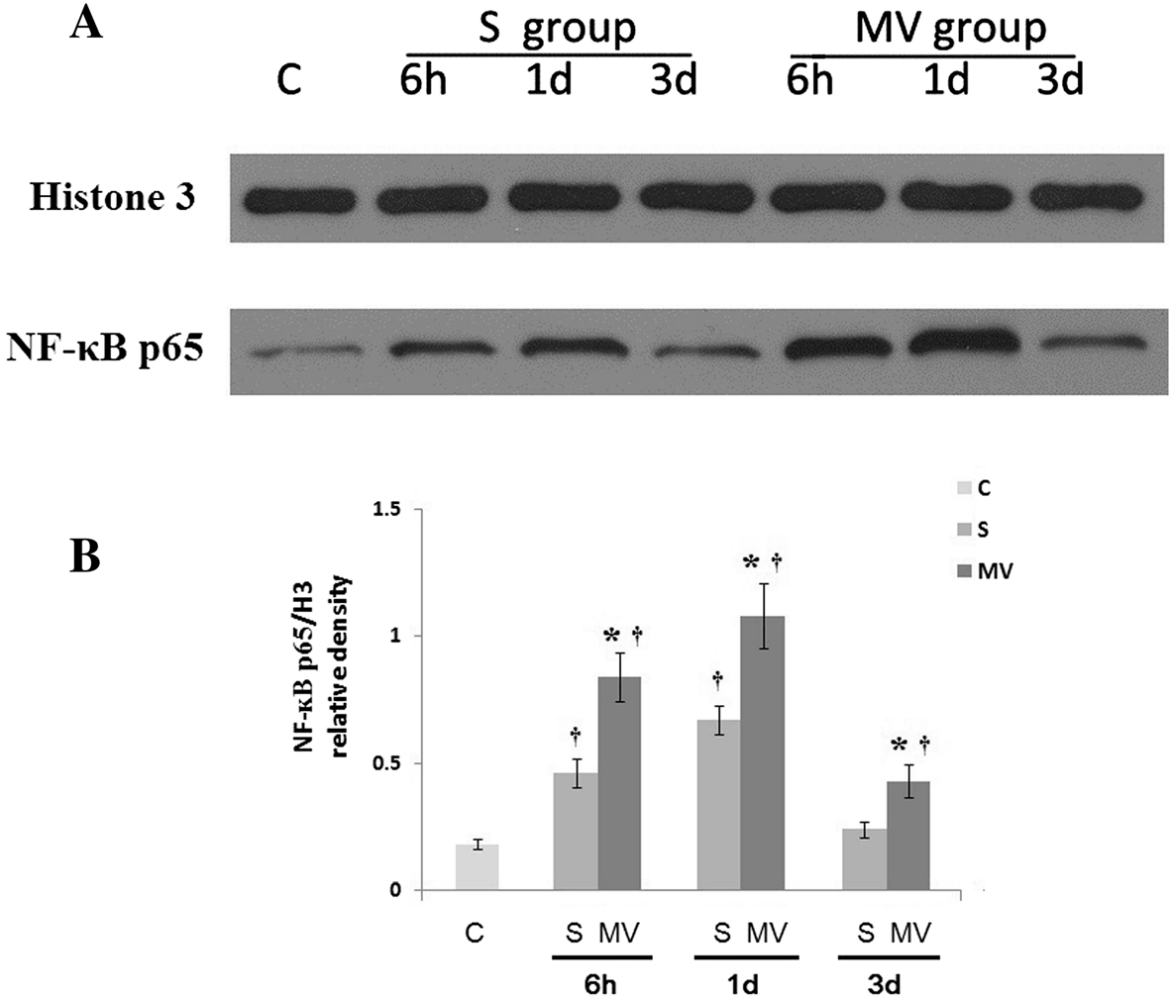
**Six-hour exposure to mechanical ventilation significantly increased nuclear factor κB activation in hippocampal tissues. (A)** Nuclear factor κB (NF-κB) p65 expression was detected by Western blot analysis. **(B)** NF-κB p65 protein expression was significantly increased in the mechanical ventilation (MV) groups compared with the surgery groups (S) at 6 hours, 1 day and 3 days postsurgery or post-MV. Western blot assay data are expressed as mean ± standard error of the mean. n =6/group. *P <0.05 compared with group S. ^†^ *P* <0.05 compared with group C.

Figure 3 shows hippocampus and plasma levels of inflammation. Six-hour exposure to MV after surgery dramatically increased the levels of IL-1β, IL-6 and TNFα in the hippocampus and plasma at 6 hours and 1 day post-MV compared with the surgery-only group (*P* <0.05) NF-κB p65 protein expression was significantly increased compared with the surgery group at any time point examined (Figure 4). However, the levels of IL-6 (Figure 3C, D) and TNFα (Figure 3E, F) in the hippocampus and plasma were not different between the MV and surgery groups on day 3 post-MV.

**Figure 5:**
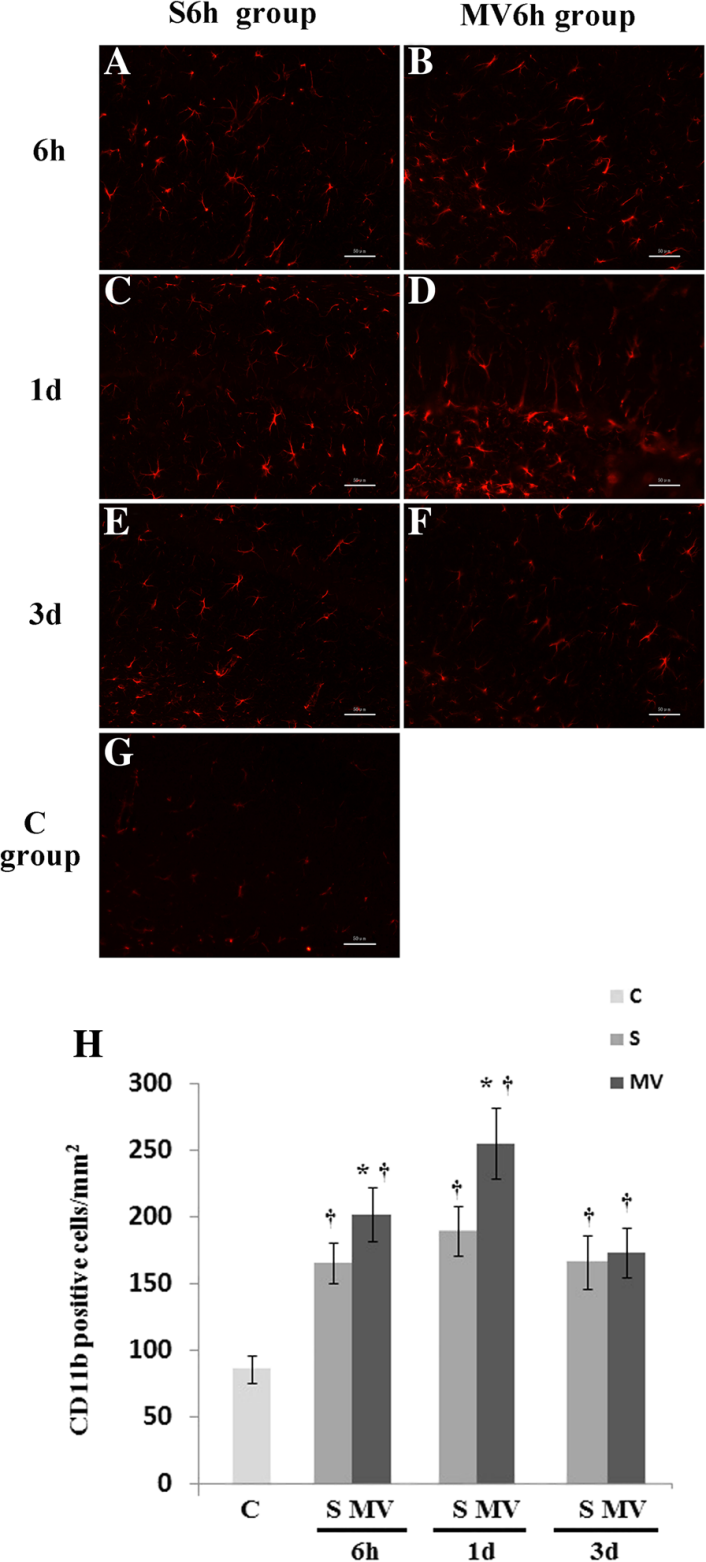
**Immunofluorescence of microglia with anti-CD11b.** Hippocampi were harvested 6 hours, 1 days and 3 days postsurgery (S) or after mechanical ventilation (MV). **(A)** through **(G)** Representative photomicrographs of tissue from only surgical animals and surgical animals treated with 6 hours of MV (pictures shown refer to CA2 region of the hippocampus in tissue. Scale bars =50 μm. **(H)** Cell counts reveal significant differences between S6h and MV6h at 6 hours and 1 day postsurgery or post-MV. One day after surgery, mice showed significantly lower levels of reactive microgliosis compared with surgical mice treated with 3 hours or 6 hours of MV. Values are mean ± SEM. n =4/group. *P <0.05 compared with group S; ^†^ *P* <0.05 compared with control group (C).

We analyzed numbers of CD11b-positive cells in the CA1, CA2 and CA3 subsections of the hippocampal formation (Figure 5). Surgery induced significant morphological changes of microglial reactivity at 24 hours compared with control animals treated only with anesthesia (*P* <0.05). The amoeboid hypertrophy of cell bodies and clumping of processes in the MV6h group seen in the entire hippocampus were more severe on day 1 (Figure 5D) than S6h group. CD11b immunoreactivity was enhanced in the hippocampus of operated animals treated with MV6h at 6 hours and 1 day post-MV (*P* <0.05).

### Ultrastructure of hippocampus

**Figure 6:**
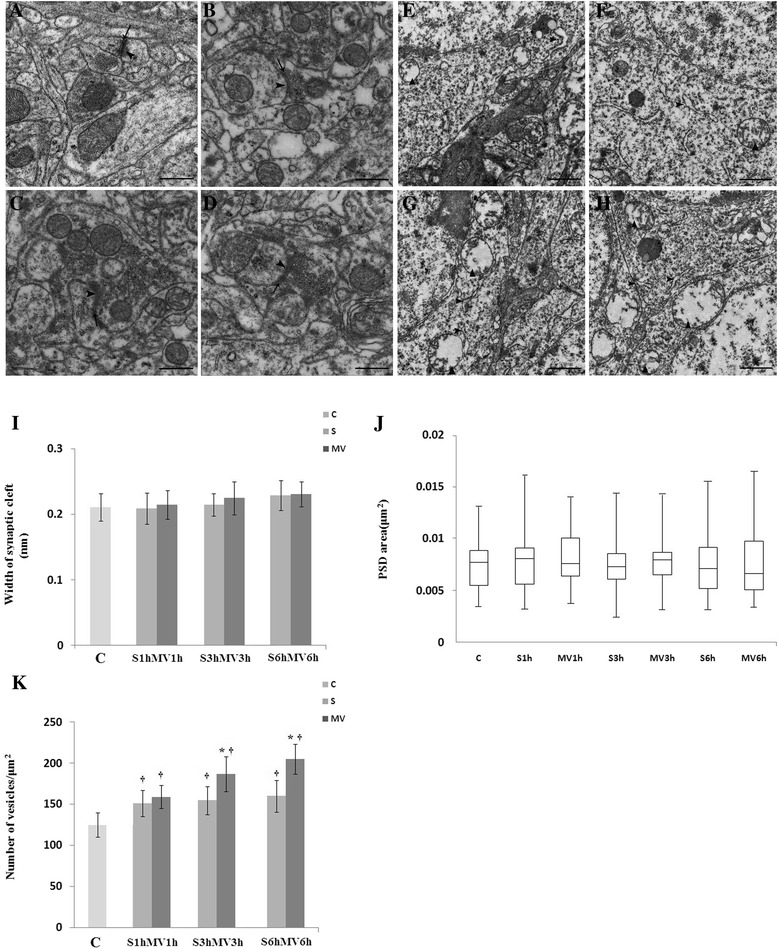
**Ultrastructural changes of synapses in the hippocampal CA1 region visualized by transmission electron microscopy.** The animals were killed at 6 hours post-MV. Six-hour exposure to mechanical ventilation (MV) aggravated impairment in the synaptic ultrastructure at 6 hours post-MV. **(A)** Control group (C). **(B)** Surgical group (S) at 6 hours (S6h). **(C)** MV group at 3 hours (MV3h). **(D)** MV group at 6 hours (MV6h). Arrows point to synaptic cleft; arrowheads point to postsynaptic density. Ultrastructural changes of the mitochondrion and endoplasmic reticulum were visualized under transmission electron microscopy. The degree of rough endoplasmic reticulum degranulation in the hippocampus was more severe in the MV6h group **(G)** and **(H)** compared with the MV3h group **(E)** and **(F)**. Scale bar =500 nm. Quantitation of the data is presented for **(I)** width of synaptic cleft, **(J)** area of postsynaptic density (PSD) and **(K)** number of vesicles. Data for each group in (J) are summarized by a box chart, in which the horizontal lines denote the 25th, 50th and 75th percentile values and the error bars denote the 5th and 95th percentile values. *P <0.05, compared with group S; ^†^ *P* <0.05, compared with group C.

To investigate the mechanism of cognitive impairment induced by only surgical animals and surgical animals treated with MV. Animals killed at 6 hours postsurgery or post-MV, the synaptic morphometric changes in the hippocampal CA1 region were observed by TEM. Degenerating presynaptic elements with a typical dark appearance and a curved PSD were observed, and these impairments of the synaptic cleft were aggravated in the MV3h and MV6h groups (Figure 6C, D). Mitochondrial swelling and vacuolation were particularly conspicuous, and the degree of rough endoplasmic reticulum degranulation in the hippocampal CA1 region was more severe in the MV3h and MV6h groups (Figure 6G, H). We measured the postsynaptic area and the width of the synaptic cleft in surgery and MV groups. As shown in Figure 6I, J and K, the number of vesicles was greater in MV6h animals than in S6h mice (*P* <0.05). However, there was no significant difference between the S1h and MV1h groups. The PSD areas and the widths of the synaptic cleft in surgical mice treated with MV were not significantly different from those of surgical mice (*P* >0.05).

### Apoptosis cascades

**Figure 7:**
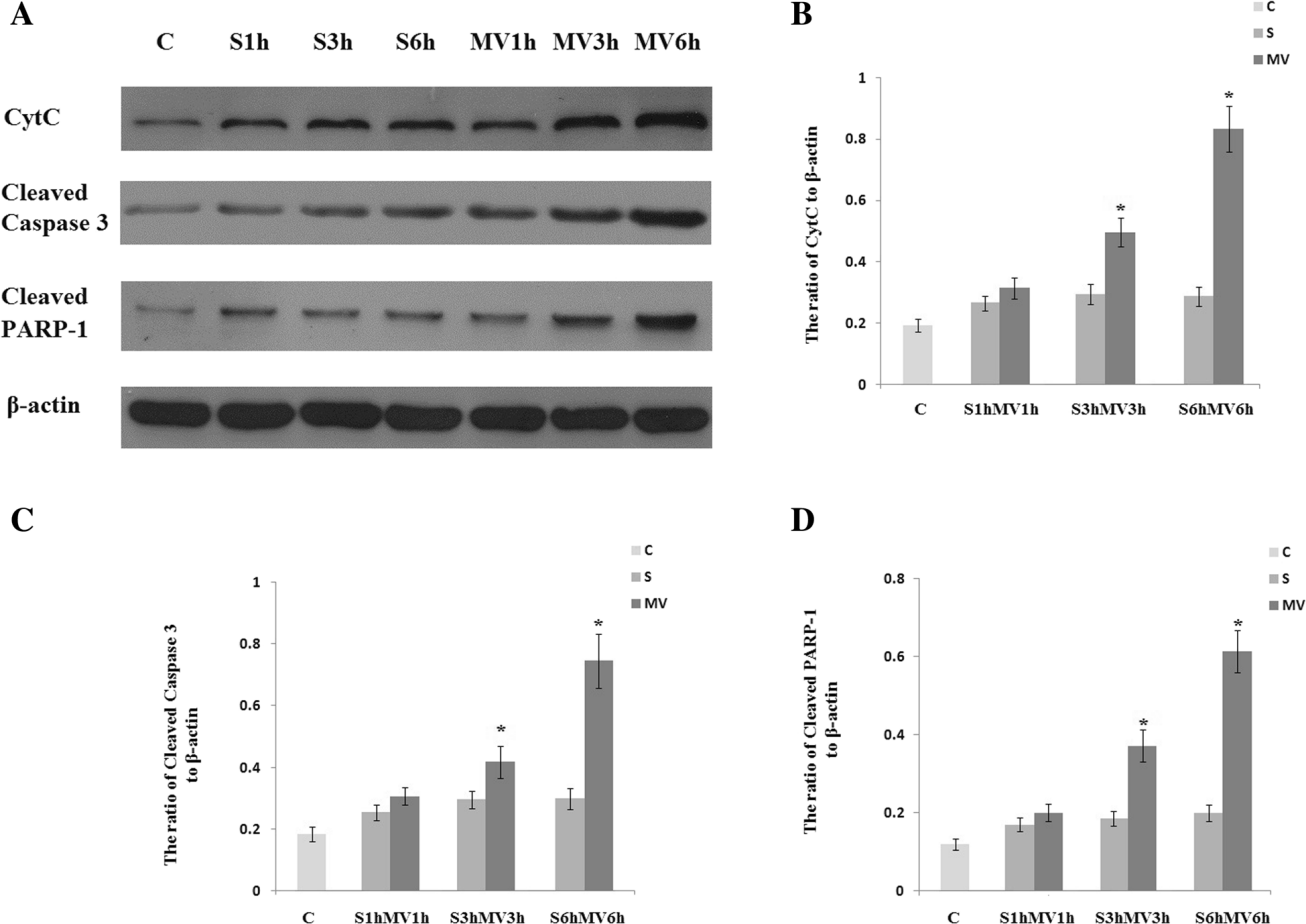
**Mechanical ventilation induced significantly greater apoptosis in surgical mice.** Six-hour exposure to mechanical ventilation (MV) induced upregulation of cytochrome *c* (Cytc), cleaved caspase-3 and cleaved poly(ADP-ribose) polymerase 1 (PARP-1) in the hippocampus after surgery (S). **(A)** Representative Western blot of Cytc, cleaved caspase-3 and cleaved PARP-1. **(B, C and D)** The results of semiquantitative analysis of the ratio of Cytc to β-actin **(B)**, the ratio of cleaved caspase-3 to β-actin **(C)** and the ratio of cleaved PARP-1 to β-actin **(D)**. All Western blot assay data are expressed as mean ± standard error of the mean. C, Control group. n =6/group. *P <0.05, compared with group S.

Caspase-3 is cleaved during the process of apoptosis, and the cleaved caspase-3 is a well-accepted biomarker for cell death by apoptosis. Cleavage of PARP-1 by caspases is also considered to be a hallmark of apoptosis. Mice ventilated with either a 3-hour or 6-hour prolonged MV strategy after surgery showed significantly elevated immunoreactivity levels for Cytc, cleaved PARP-1 and cleaved caspase-3 in the hippocampus at 6 hours post-MV compared with surgery group mice (Figure 7).

## Discussion

We found that MV6h induced cognitive decline following surgery and increased activation of microgliosis and apoptotic cascades after surgery, thus supporting the hypothesis that detrimental effects of prolonged MV after surgery may affect the brain. Lungs can “sense” long-term mechanical stimuli by lung mechanoreceptors that can communicate this information to the brain [15]. On this background of prior surgery, MV-induced inflammation and apoptotic cascades could be among the most common triggers. These results provide novel and important data that might have clinical relevance during the management of surgical patients.

The ventilatory strategy may affect the CNS by altering the inflammatory response at the lung level and then altering neurotransmission in the brain [16]. We focused our study on prolonged MV-induced postoperative memory dysfunction involved in systemic inflammatory and neuroinflammatory responses. IL-1β mediates part of the inflammatory response to both infection and injury [17]. In addition, it is likely to have a prominent role in postoperative cognitive dysfunction (POCD). It is well established that attenuation of the IL-1β response to surgery prevents postoperative memory dysfunction [18]. On the basis of this prior work suggesting a link between inflammation in the hippocampus and memory impairment, we assessed activated microglia in subregions of the hippocampus. In our study, with 6-hour exposure to MV after surgery, hippocampal inflammation was demonstrated by a local increase in the expression of IL-1β, IL-6 and TNFα as well as reactive microgliosis. MV triggered systemic inflammatory responses after surgery, and prolonged MV may affect the CNS by altering the systemic inflammatory response [19].

A possible causal relationship between MV, inflammation and memory impairment was suggested by prolonged MV that resulted in increased surgery-induced peripheral and hippocampal cytokine expression, apoptotic cascades and reactive microgliosis. In this case, AS surgical trauma provokes a neuroinflammatory response, surgical intervention induces systemic cytokine release that is followed by hippocampal inflammation and memory impairment. The apoptotic cascades quickly induced by postoperative ventilation might be associated with hippocampal inflammation induced surgical intervention. Within the hippocampus, these activated microglia release proinflammatory cytokines that are capable of attenuating the long-term potentiation that is the neurobiologic correlate of learning and memory [20]. Microglial reactivity could be both the cause and the effect of the increased IL-1β hippocampal expression in the open reduction and internal fixation model [18]. It is also likely that microglial cells are responsible for local production of IL-1β. IL-1β and activated microglia have been demonstrated to impair long-term potentiation [21], which is associated closely with memory formation [22].

To explore the molecular mechanism explaining of prolonged MV after surgery, we investigated the activity of NF-κB, a key regulatory protein for inflammation. Within the peripheral macrophages, NF-κB is activated to enhance transcription and subsequent synthesis and release of proinflammatory cytokines, including TNFα, high-mobility group box 1 protein and IL-1β [23]. Cognitive deficits and blood–brain barrier damage were associated with early induction of TNFα and Fas mRNA and/or protein, NF-κB-binding activity and activation of microglia and astrocytes [24]. We showed that NF-κB activation is induced by MV following surgery, and this induction may explain some of the neuroinflammatory effects of MV.

Because mitochondrial swelling after MV6h following surgery was significantly enlarged, we were interested to learn whether there was an increase in the release of Cytc and activation of apoptotic cascades. The mitochondrial apoptosis pathway is a major apoptosis pathway, and a large number of apoptosis-related proteins are found in mitochondria, including Cytc, apoptosis-inducing factor and procaspases 3, 8 and 9 [25]. Caspase-3 can cleave many substrate proteins, such as PARP. Overactivation of PARP after cleavage by caspase-3 leads to DNA injury and subsequently to apoptotic cell death [26]. The activation of apoptotic cascades was observed in the MV6h group, thus suggesting that mechanically induced stress in the lung after surgery could promote impaired memory through association with hippocampal inflammation, which deserves being explored in further investigations.

Work at several laboratories has suggested that an inflammatory component could contribute to impaired memory [27,28]. In the present study, we trained animals prior to surgery or MV. This allowed us to remove the surgical influence of the acquisition phase on assessment of memory postoperatively. We found no differences between the MV1h and S1h groups at any time point examined. It is likely that properly regulated MV exposure time was required for memory impairment.

### Limitations of the study

Despite considerable medical and economic burdens, delirium is poorly understood, and a lack of animal models has contributed to this. Therefore, the behavior assessment of cognitive decline in this study does not allow us to conclude whether postoperative delirium (POD) or POCD is mechanistically linked. POD is a common neuropsychiatric syndrome characterised by acute deterioration and fluctuations in postoperation mental status. However, POCD is characterized by long-term cognitive including memory impairment persisting at least three months after a major surgical intervention. After 6-hour exposure to MV following surgery, physiological cycles of mice entered the stage of sleep. The lack behavioral results data from continuous monitoring within 24 hours confirms fluctuating impairment of consciousness. Further studies with possible targeted, prolonged MV increasing the occurrence of POD would be needed to establish this.

Animal models of complex diseases are potentially limited by interspecies differences. Longer duration of MV stimulation could lead to increased mortality in the surgical model of mice. Moreover, as this was a short-term study focused on the acute exaggeration phase of neuroinflammation, we are unable to extrapolate the long-term effects from the data.

### Clinical relevance

The etiology of cognitive impairment in critically ill patients is undoubtedly multifactorial and is the subject of ongoing discussion [29]. Among different risk factors identified in observational studies, MV is one of the most relevant. The inflammatory response in the lungs due to MV may lead to distal organ dysfunction, including the brain [30]. Nevertheless, inflammatory mechanisms of MV-induced postoperative memory decline that is reflected in POD and POCD are poorly understood. Few studies have explored the influence of prolonged MV after surgery on neuroinflammation. Our findings regarding prolonged MV resulting in increasing peripheral and hippocampal cytokine expression, reactive microgliosis, apoptotic signals triggering caspase activation and behavioral impairment might have implications for understanding how POD or POCD happen in the intensive care unit.

## Conclusions

In summary, neuroinflammation is associated with full maintenance of memory following postoperative prolonged MV. The work reported here shows that MV-induced inflammation disrupted hippocampal memory consolidation, as evidenced by reduced contextual freezing time exhibited by mice that underwent MV after surgery. Our data suggest that prolonged MV further aggravates cognitive decline after surgery that may stem from upregulation of hippocampal IL-1β, IL-6 and TNFα, partially via activation of gliocytes in surgical mice.

## Key messages

- Prolonged mechanical ventilation might affect postoperative memory dysfunction in surgical mice and might be associated with neuroinflammation.
- This hypothesis is supported by evidence that detrimental effects of prolonged mechanical ventilation after surgery may affect the brain.
- Six-hour exposure to MV following surgery might play a synergistic role in hippocampal IL-1β, IL-6 and TNFα expression, as well as activation of gliocytes.
- We addressed an emerging area of research by studying mechanical ventilation experimentally in surgical mice to determine how the cognitive deficits expressed as POD happen.

## Abbreviations

CNS: Central nervous system
Cytc: Cytochrome *c*
FC: Fear conditioning
FiO_2_: Fraction of inspiration oxygen
IL: Interleukin
MV: Mechanical ventilation
NF-κB: Nuclear factor κB
PARP: Poly(ADP-ribose) polymerase
POCD: Postoperative cognitive dysfunction
POD: Postoperative delirium
PSD: Postsynaptic density
TEM: Transmission electron microscopy
TNFα: Tumor necrosis factor α
